# Understanding the Effectiveness of Natural Compound Mixtures in Cancer through Their Molecular Mode of Action

**DOI:** 10.3390/ijms18030656

**Published:** 2017-03-17

**Authors:** Thazin Nwe Aung, Zhipeng Qu, R. Daniel Kortschak, David L. Adelson

**Affiliations:** 1 Department of Genetics and Evolution, School of Biological Sciences, The University of Adelaide, Adelaide, South Australia 5005, Australia; thazin.nweaung@adelaide.edu.au (T.N.A.); zhipeng.qu@adelaide.edu.au (Z.Q.); dan.kortschak@adelaide.edu.au (R.D.K.); 2 Zhendong Australia China Centre for Molecular Chinese Medicine, The University of Adelaide, Adelaide, South Australia 5005, Australia

**Keywords:** cancer, apoptosis, chemosensitization, microRNA, natural compound mixtures, metal derivatized natural compounds

## Abstract

Many approaches to cancer management are often ineffective due to adverse reactions, drug resistance, or inadequate target specificity of single anti-cancer agents. In contrast, a combinatorial approach with the application of two or more anti-cancer agents at their respective effective dosages can achieve a synergistic effect that boosts cytotoxicity to cancer cells. In cancer, aberrant apoptotic pathways allow cells that should be killed to survive with genetic abnormalities, leading to cancer progression. Mutations in apoptotic mechanism arising during the treatment of cancer through cancer progression can consequently lead to chemoresistance. Natural compound mixtures that are believed to have multiple specific targets with minimal acceptable side-effects are now of interest to many researchers due to their cytotoxic and chemosensitizing activities. Synergistic interactions within a drug mixture enhance the search for potential molecular targets in cancer cells. Nonetheless, biased/flawed scientific evidence from natural products can suggest false positive therapeutic benefits during drug screening. In this review, we have taken these factors into consideration when discussing the evidence for these compounds and their synergistic therapeutic benefits in cancer. While there is limited evidence for clinical efficacy for these mixtures, in vitro data suggest that these preparations merit further investigation, both in vitro and in vivo.

## 1. Introduction

Cancer remains one of the highest causes of death globally. Various types of chemotherapies fail due to adverse reactions, drug resistance, and target specificity of some types of drugs. There is now emerging interest in developing drugs that overcome the problems stated above by using natural compounds, which may affect multiple targets with reduced side effects and which are effective against several cancer types. Natural compounds from various sources including plants, animals, and microorganisms offer a great opportunity for discovery of novel therapeutic candidates for the treatment of cancer [1]. Apoptosis is a self-destructive programmed sequence of signal transduction events that destroys cells that become a threat, or are no longer necessary to the organism [2]. When there is aberrant apoptosis, cells that should be killed instead become immortal, leading to the pathogenesis of many diseases including cancer. 

Apoptosis falls into two categories: extrinsic and intrinsic apoptosis. Extrinsic apoptosis occurs when cell death is triggered by binding of extracellular stress ligands to transmembrane receptors such as death receptors CD95 (APO-1/Fas) and tumor necrosis factor (receptor1) [3] as well as dependence receptors such as netrin-1 receptor UNC5H4 [4,5]. In contrast, intrinsic apoptosis occurs in the mitochondria through heterogeneous signaling cascades dependent or independent of caspases [4]. Failure to trigger complete apoptosis in the unhealthy cell population is a cause for cells to grow out of control, leading to cancer [6]. When apoptosis is defective in one of the main apoptotic pathways, it increases the likelihood of the cell becoming cancerous. Various well-established treatments have been designed to destroy cancer cells through apoptosis. Another important mechanism of cell death in cancer cells in response to chemotherapy is autophagy, which takes places in the lysosome by self-degrading intracellular proteins and organelles. It triggers cell death in the absence of apoptotic regulators, but in the presence of important autophagy-regulated genes such as *BECN1* [7] and *ATG5* [8]. In this review, we primarily confine our discussion to apoptotic cell death and autophagic cell death caused by natural chemotherapeutic agents in the context of cancer.

Resistance to treatments that target apoptotic cell death is indicative of treatment failure. Anti-apoptotic mutations during cancer progression reduce chemotherapy-induced apoptosis in spontaneous murine tumors [9] and produce multi-drug resistance [10]. Therefore, understanding how to induce cell cytotoxicity via chemosensitization is as important as how to trigger apoptosis in cancer cells with chemotherapies. It has been reported that natural compounds such as quercetin [11] and tetrandrine [12], known to have anti-tumor activities, are able to not only kill cancer cells but also restore drug sensitivity [13,14]. Moreover, there is evidence that natural compounds including rhamnetin and cirsiliol can radiosensitize in non-small cell lung cancer (NSCLC) [15]. This suggests that natural compounds can have therapeutic effects in cancer chemo-radiotherapy. 

Effective development of an anti-cancer drug needs to consider different sets of upregulated, downregulated, and mutated genes and their regulatory pathways in cancer cells. Computational genomics is a powerful tool to identify differential gene expression based on cancer treatment, as it improves our understanding of challenging mechanistic changes in cancer cells and facilitates treatment with a wide range of molecular targets. Whole transcriptome sequencing comprehensively investigates messenger RNA (mRNA)-Seq and small/non-coding RNA-sequencing (RNA-Seq), analyzing tens of thousands of RNA transcripts to uncover their genetic functions. Transcriptomic results subjected to Gene Ontology (GO) clustering and annotation identify differentially expressed genes and can further identify candidate target pathways [16]. Here we highlight the efficacy of complex natural compound mixtures by using molecular approaches with specific emphasis on cancer apoptosis and chemosensitization.

## 2. Treatment of Cancer through Targeting Apoptosis

There are many therapies for treating cancer, including surgery, radiation therapy, hormone therapy, chemotherapy, and targeted therapies such as immunotherapy and monoclonal antibody therapy. Depending on the type of cancer and underlying biological conditions in the patient, therapy consists of either a single or combination of classical treatments such as surgery, chemotherapy, and/or radiotherapy. 

Chemotherapy is a treatment that uses anti-cancer drugs to damage DNA in unhealthy and rapidly dividing cancer cells. Chemotherapy with a defined dosage is usually used to trigger cancer cell cytotoxicity at desirable apoptotic rates. The effectiveness of chemotherapeutic agents depends on their type, dosage, and any adverse reactions in patients. There are several anti-cancer drugs used alone or in combination with other agents to kill cancerous cells. Chemotherapeutic drugs that include synthetic, semi-synthetic, and naturally occurring compounds are cytotoxic, and can destroy both cancerous cells and rapidly dividing normal cells. These agents signal through both death receptors and mitochondrial pathways to induce one or more of the apoptotic pathways [17]. They are characterized based on their structure, derivation, and mechanism of action. Some affect parts of the cell cycle, while others are not phase specific. Depending on the mechanism of action, they are categorized into different groups including alkylating antineoplastic agents, kinase inhibitors, vinca alkaloids, anthracyclines, antimetabolites, aromatase inhibitors, and topoisomerase inhibitors [18]. Nonetheless, the pharmacokinetic variability of synthetic drugs in patients often limits optimal effectiveness with minimal toxic side effects. On the other hand, treatment of cancer by natural compounds and their semi-synthetic analogues both in vitro and in vivo shows promising results against different malignancies [19,20]. Natural compounds such as sesquiterpenes, flavonoids, alkaloids, diterpenoids, saponins, and polyphenolic compounds [11,21] can be substituted for, or applied in combination with, existing drugs.

## 3. Natural Compounds as Anti-Cancer Agents

Natural compounds with potent anti-cancer activities are widely available from different plant tissues. Eighty percent of the population worldwide traditionally use natural compounds contained in medicinal plants [22] and are largely dependent on them. Naturally occurring compounds target tumor cells by regulating cell death pathways such as extrinsic and intrinsic apoptosis pathways and autophagic pathways. Evidence from in vitro and in vivo studies in prostate cancer treatment with isoflavones and phytoestrogens from soy showed NF-κB deactivation, apoptosis induction, and angiogenesis inhibition [23,24]. A collection of plant-derived natural anti-cancer compounds can be found at Naturally Occurring Plant-based Anti-cancer Compound-Activity-Target Database (NPACT, http://crdd.osdd.net/raghava/npact/) where approximately 1980 experimentally validated compound-target interactions are documented [25]. Millimouno et al. also reviewed promising natural compounds and their related natural sources, pharmacological actions, and molecular targets in details [21]. 

## 4. Traditional Chinese Medicines (TCMs) as Anti-Cancer Agents

Due to the complex etiology and pathophysiology of cancer, it is relatively difficult to treat the disease with just single target drugs. Moreover, regardless of the specificity and efficiency of single target therapy, it is difficult to achieve optimal cytotoxic effects on cancer cells because of their rapid molecular adaptations. In contrast, synergistic interactions within multi-component drug preparations allow us to broaden the search for potential molecular targets in cancer cells. Traditional Chinese Medicines (TCM) is formulated based on the compatibility and interrelationships between herbal ingredients that render synergistic therapeutic benefits [26]. TCM uses a combinatorial approach where the application of two or more agents at their respective effective concentrations achieves a synergistic effect that boosts cytotoxicity to cancer cells and can have additional effects on the tumor environment and the immune response to tumors. Therefore, TCM has been used as an alternative or complementary medicine worldwide, and has long been used to treat cancer in China. Chinese herbal medicinal products have been used for cancer prevention and treatment for many years [27], and there is evidence to suggest that TCMs are effective against cancer recurrence and metastasis and can enhance quality of life (QoL), and prolong survival time [27]. For instance, Bioactive polysaccharides with β-1,3, β-1,4, and β-1,6 side branches in TCM stimulate the immune system, thereby indirectly suppressing tumors [28]. TCM is also used to reduce the side effects of conventional chemotherapy for advanced pancreatic cancer, advanced colorectal cancer, and breast cancer [29,30,31]. There are a range of TCM extracts from *Anemarrhena asphodeloides*, *Artemisia argyi*, *Commiphora myrrha*, *Duchesnea indica*, *Gleditsia sinensis*, *Ligustrum lucidum*, *Rheum palmatum*, *Rubia cordifolia*, *Salvia chinensis*, *Scutellaria barbata*, and *Uncaria rhychophylla* that specifically inhibit cancer cell proliferation from breast, lung, pancreas, and prostate tissues of human and mouse, but show limited inhibition against normal human mammary epithelial cell growth [32]. Artemisinin derivatives artesunate (ART) and dihydroartemisinin have been shown to inhibit cancer cell proliferation and suppress angiogenesis in cervical, uterus chorion, embryo transversal cancer, and ovarian cancer [33]. Because naturally occurring compounds such as plant extracts in TCM are highly chemically diverse, they have become highly significant in the discovery and development of effective therapeutic anti-cancer drugs. TCM preparations can contain alkaloids, flavonoids, saponins, terpenes, polyphenols, fatty acids, and essential oils as bioactive ingredients [34,35]. 

### 4.1. Natural Compounds from TCM as Cancer Therapeutics

The main components of TCM such as alkaloids, flavonoids, and saponins are used either individually or as mixtures to treat different types of cancer. Below we list compounds contained in various TCMs that have been found to have anti-cancer activities, including triggering apoptosis.

### 4.2. Alkaloids

Alkaloids are more abundantly found in broad ranges of the plant kingdom than other classes of natural plant products [36] and are active against various cancers. Alkaloids commonly consist of a nitrogen atom within a heterocyclic ring [37] and are of relatively low toxicity. Several alkaloids have a wide range of significant biological functions including anti-inflammatory, anti-bacterial, anti-diabetic, and anti-cancer activities [38,39,40]. Some well-developed semi-synthetic anti-cancer drugs are alkaloid derivatives including vinblastine, vinorelbine, vincristine, and vindesine. They are the most important active ingredients in traditional medicine and have been approved for cancer treatment in the United States and Europe [41]. 

Matrine is a major quinolizindine alkaloid found in the *Sophora flavescens* Aiton plant [42]. Matrine stimulates major apoptotic cascades by upregulating Fas/FasL and Bax, and downregulating Bcl-2 leading to the activation of caspase-3, -8, and -9 in MG-63, U-2OS, Saos-2, and MNNG/HOS human osteosarcoma cells [43]. It also represses cancer metastasis via vascular endothelial growth factor (VEGF)-Protein Kinase B (Akt)-nuclear factor kappa-light-chain-enhancer of activated B cells (NF-κB) signaling in MDA-MB-231 breast cancer cells. The reduction of Bcl-2/Bax protein and mRNA levels by matrine leads to an increase of cell cycle arrest in cancer cells [44]. In human medulloblastoma D341 cells, increased expression of Bcl-2 and decreased expression of Bax is triggered by matrine through caspase-3 and -9 mediated apoptotic pathways [45]. In HepG2 cells, matrine induces tumor suppressor transcription factor p53 through the adenosine monophosphate-activated protein kinase (AMPK) signal transduction pathway, resulting in autophagic cell death through the p53/AMPK signaling pathway [46]. Interestingly, this research reported that downregulation of AMPK leads to a switch to apoptotic cell death from autophagic cell death [46]. The sequential signal transduction leading from autophagy to apoptosis via the activation of p53 was discussed by Guillermo Mariño et al. [47]. Furthermore, metabolomics analysis of matrine treated HepG2 cells identified lipid droplet metabolites, which are substrates for macro autophagy that may partly drive immunity and apoptosis [48]. Li et al. also reported that matrine treatment reduced the level of glutathione (GSH), and the elevated level of GSH is related to chemoresistance in cancer [49]. The above results provide evidence that matrine alone can induce cell death and can be effective against various tumor types. 

Oxymatrine is another major quinolizindine alkaloid found in the *Sophora flavescens* Aiton plant. It is cytotoxic to SW1116 human colon cancer cells by downregulating human telomerase reverse transcriptase (hTERT) and upregulating *p53* as well as *mad1* in a concentration dependent manner [50]. It also inhibits the growth of GBC-SD and SGC-996 gallbladder cancer cells via the activation of caspase-3 together with Bax and the suppression of Bcl-2 and NF-κB [51]. It is also known that a mixture of oxymatrine and micellar nanoparticles is an effective proliferation inhibitor of SMM7721 cells [52]. Oxymatrine treatment significantly induces apoptosis by increasing Bax protein expression and reducing Bcl-2 in human lung cancer A549 cells [53]. Proteomic analysis has shown that oxymatrine induces apoptosis in HeLa cells by inhibiting inosine monophosphate dehydrogenase type II (IMPDH2), mitochondrial related apoptotic protein [54]. These studies suggest that oxymatrine may be a useful drug candidate for cancer therapy. 

Another type of natural alkaloid is tetrandrine, contained in the Chinese medicinal plant Hang-Fang-Chi, *Radix Stephania tetrandra* S. Moore. This compound has anti-inflammatory, immunosuppressive, and anti-cancer activities [55]. It was reported that a derivative (H1) of tetrandrine has the ability to reverse multi-drug resistance [56]. It inhibits cancer cell proliferation and induces apoptosis in human esophageal cancer cell lines ECa109, Eca109-C3, and human monoblastic leukemic U937 cells. It is also effective in reversing multi-drug resistance in Adriamycin-resistant human breast cancer MCF-7/Adr and human nasopharyngeal cancer KB_v200_ [12]. The molecular mechanisms of action of tetrandrine in cancer cells include upregulation of Bax, Bak, Bad, and apaf-1, downregulation of Bcl-2 and Bcl-xl, releasing cytochrome *c*, and activation of caspase-3 and -9 in the apoptotic mitochondrial pathway [56]. The efficacy of tetrandrine with respect to activation of the intrinsic apoptosis pathway highlights its potential importance as a therapeutic agent. 

The semi-synthetic alkaloid analogue vinblastine is an anti-mitotic drug that was originally isolated from the periwinkle plant *Catharanthus roseus* (L.) G. Don. It kills cancer cells by shortening microtubules, disrupting microtubule function resulting in the disappearance of the mitotic spindle, thereby inhibiting cell proliferation [57]. Low concentrations of vinblastine have been shown to slow down or block mitosis in HeLa and BSC cells [57]. Vinblastine is highly potent in relapsed/refractory anaplastic large-cell lymphoma (ALCL) with 65% five-year overall survival [58]. 

### 4.3. Flavonoids

Flavonoids are plant secondary metabolites, widely present in fruits and vegetables that are consumed daily. They generally have a sixteen-carbon skeleton and the structures vary around the heterocyclic oxygen ring [59]. Research has shown that flavonoids inhibit cell proliferation and angiogenesis, cause cell cycle arrest, induce cell apoptosis, and reverse multi-drug resistance and/or a combination of the aforementioned mechanisms [60]. 

Trifolirhizin, a pterocarpan flavonoid, is present in the *Sophora flavescens* Aiton plant. It was shown that trifolirhizin reduces the expression of pro-inflammatory cytokines such as TNF-α, cyclooxygenase-2 (COX-2) and IL6 in experimentally lipopolysaccharide (LPS)-stimulated mouse J774A.1 macrophage [61]. Zhou et al. also showed that it was able to inhibit the growth of human ovarian A2780 and lung H23 cancer cells in vitro. The compound also has anti-proliferative activity in oral carcinoma SCC2095 cells [62]. A combination of trifolirhizin together with maackiain (a constituent of *Trifolium pratense*) has been shown to induce apoptosis in human leukemia HL-60cells. This mixture of compounds resulted in the degradation of DNA into oligonucleosome-size fragments in a time- and dose- dependent manner [63]. These results suggest that trifolirhizin may possibly be developed as an anti-inflammatory nutraceutical for cancer prevention as well as for the mitigation of DNA damage through apoptosis.

Curcumin is a traditional medicine and main curcuminoid of *Curcuma longa* and has been implicated in the perturbation of several genetic pathways [64,65]. It was reported that it selectively targets tumor cells rather than normal cells in vitro and activates different apoptotic pathways including caspase, induction of death receptors and DNA fragmentation, mitochondrial activation, autophagy pathways, inhibition of NF-κB, and inhibition of COX-2 and 5 LOX [65]. In proteomic identification of curcumin treated MCF-7 cells, 3-PGDH and ERP29 were found to be upregulated and TDP-43, SF2/ASF, and eIF3i were downregulated, suggesting curcumin induced apoptosis in breast cancer [64]. However, due to its lack of aqueous solubility, high concentrations are required to demonstrate potential chemotherapeutic efficacy [66]. While many researchers are optimistic regarding curcumin’s potential effectiveness against cancer, there is evidence to show that curcumin has no therapeutic benefits despite many published articles and clinical trials [67]. Skepticism about curcumin is based on both poor characterization of curcumin and pan-assay interference in many experiments that indicated that curcumin was a promising compound for cancer treatment. Yet, in spite of no significant effects in trials, researchers still think that because curcumin can interact with many proteins and because of suggestive trends in trial results, there is still justification for further study [68]. Until better experiments are carried out, the anti-cancer activity of curcumin remains unconfirmed. 

A flavonoid, quercetin, is abundant in daily-consumed foods such as onions (*Allium cepa*) with a wide range of anecdotally reported health benefits that include anti-oxidant, anti-inflammatory, and anti-cancer activities in vitro and is effective against various cancer cells [69]. Quercetin mainly targets the cell cycle at G1/S and G2/M check points by inducing the p21 CDK inhibitor while decreasing pRb phosphorylation, thereby blocking E2F1, which is an important transcription factor of DNA synthesis proteins [70]. Deng et al. reported that apoptosis-mediated cell death from quercetin treatment resulted from arresting the cell cycle at G0/G1 phase in MCF-7 breast cancer cells. They also showed that increasing concentrations of quercetin were directly proportional to the decreasing concentrations of survivin, a member of a protein family that negatively regulates apoptosis [71]. Proteomic analysis revealed that quercetin treatment suppressed cell proliferation while arresting mitosis leading to apoptosis by downregulating IQGAP1 and β-tubulin and their interactions with other proteins in HepG2 cells [72]. Despite the abundance of quercetin, it has not been investigated in cancer clinical trials. 

### 4.4. Saponins

Saponins are found not only in a wide range of plants but also in animals, and have different carbon backbones that classify them as either steroids or triterpenes. They are secondary metabolites with potent biological functions. These compounds are active against several tumors not only as single compounds but also in combination with conventional therapies by causing cell cycle arrest and triggering apoptosis [73]. 

Chikusetsusaponin IVa butyl ester (CS-IVa-Be) is an apoptotic triterpenoid saponin extracted from *Acanthopanas gracilistylus* herb. The extract from this Chinese medicinal herb has been found to cause cell cycle arrest at G0/G1 stage in a variety of cancer cell lines including MT-2, Raji, HL-60, TMK-1, and HSC-2 [74]. The compound induces apoptosis in MDA-MB-231 cells by inhibiting IL-6 family induced STAT3 activity through the IL-6/JAK/STAT3 signaling pathway. It also sensitizes the Tumor necrosis factor (TNF)-related apoptosis-inducing ligand (TRAIL), a specific inducer of cancer cell apoptosis, in TRAIL resistant MDA-MB-231 cells by upregulating death receptor 5 (DR5) [75]. Because of this, CS-IVa-Be induces apoptosis upon treatment with TRAIL in TRAIL resistant MDA-MB-231 cells. 

Polyphyllin D is a promising anti-proliferative steroidal saponin extracted from the traditional Chinese medicinal plant *Paris polyphylla*. The cytotoxic activity of polyphyllin D was observed via induction of DNA fragmentation and dissipation of mitochondrial membrane potential ∆ψm, resulting in mitochondrial dysfunction and loss of membrane integrity in MCF-7 and MDA-MB-231 cells [76]. A 50% reduction in tumor growth from 10 consecutive days of polyphyllin D administration in mice was also documented. 

Diosgenin is effective against HCT-116 human colon cancer cells by reducing both mRNA and protein expression of 3-hydroxy-3-methylglutaryl CoA reductase, resulting in apoptosis [77]. It truncated the poly (ADP-ribose) polymerase protein from 116-kDa to an 85 kDA fragment, which leads to the induction of apoptosis. This indicates that Diosgenin is a potent apoptosis inducer in HCT-116 cancer cells. Diosgenin arrested the cell cycle at sub-G1 phase, suppressed FAS expression, and inhibited mammalian target of rapamycin (mTOR) phosphorylation in HER2 overexpressing human AU565 breast cancer cells, inhibiting cell proliferation [78]. 

Another apoptotic triterpenoid saponin is Macranthoside B (MB), extracted from *Lonicera macranthoids.* It is strongly effective in various tumors via mitochondrially mediated apoptosis resulting from an increased Bax/Bcl-2 ratio [79]. Furthermore, MB induced apoptosis via autophagy through the ROS/AMPK/mTOR pathway, while elevating reactive oxygen species (ROS) together with 5′ AMPK, and reducing mTOR in human ovarian cancer A2780 cells [80]. 

With respect to the cytotoxic properties of saponins, a wide range of these compounds has been tested, with some shown to be potent apoptotic inducers. Yet the potential of this class of compounds remains to be fully explored, and they may also be effective in combination with other agents to synergistically enhance their therapeutic effect in cancer. 

### 4.5. Drugs Based on Mixtures of Compounds

Compound Kushen Injection (CKI), approved by the State Food and Drug Administration of China, has been used to treat different types of cancer, including liver, gastric, and non-small cell lung carcinoma in combination with Western anti-cancer agents [81]. It contains alkaloids, flavonoids, saccharides, and organic acids [82] and is extracted from two medical herbs including *Radix* *Sophorae flavescentis* and *Rhizoma Smilacis Glabrae* [83]. It modulates immunity, decreases inflammation, relieves cancer pain, and, most importantly, has anti-neoplastic activity [83]. For example, CKI downregulates β-catenin through the Wnt signaling pathway, which in turn targets the oncogenes *c-MYC* and *CyclinD1* [84], leading to suppression of MCF-7 cancer stem cell-like side population (SP) cells [85]. A systematic review and meta-analysis reported that CKI could reduce adverse effects in cancer patients and improved total pain relief and QoL [86]. Transcriptome analysis of CKI treated MCF-7 cells by Qu et al. revealed that the mixture inhibited cell proliferation and induced apoptosis in a concentration-dependent manner by primarily targeting the cell cycle in MCF-7 cells [87]. Qu et al. also showed that long non-coding RNA (lncRNA) *H19* was dramatically downregulated in MCF-7 cells treated with CKI [87]. *H19* is overexpressed in several cancer types and is associated with tumor metastasis, for example, where lncRNA *H19* suppresses miR-630, perturbing the inhibition of EZH2 in nasopharyngeal carcinoma [88]. The primary effect of CKI on cancer cells is through the cell cycle, but it also affects many other pathways and it may be useful as both an anticancer and anti-inflammatory agent. It was claimed that CKI is effective in inhibiting metastasis and reversing multi-drug resistance (MDR) as well [83]. Yet, there is currently no research evidence supporting the effectiveness of CKI on reversing MDR in English-language journals. Therefore, in vivo and clinical relevance of the drug should be researched to establish the effective usage of CKI with respect to chemosensitizing activities. 

Anti-tumor B (ATB), known as Zeng Sheng Ping, is also an herbal medicine which is formulated from six different medicinal plants, and its main constituents are flavones, alkaloids, phytosterols, sapogenins, triterpenes, and triterpenoids [89]. ATB decreased lung tumor load by approximately 60% in both wild-type and Ink4a/Arf tumor suppressor gene-deficient mice and 90% in p53 transgenic mice [89]. The drug markedly reduced cell proliferation by inhibiting the mitogen-activated protein kinase (MAPK) pathway while increasing apoptosis by reducing Bcl-2 in oral cancer in hamsters [90]. Lim et al. also showed that the Notch2 receptor, which is an important signaling regulator of brain tumors, and its downstream effector gene *Hes1* were downregulated by ATB [91]. ATB induced apoptosis in both U87 glioblastoma and DAOY medulloblastoma cells [91], suggesting that ATB might be effective against multiple tumors. Based on the results from animal models, ATB has shown chemo-preventive activities in hamsters and mice with carcinogen-induced oral cancers [92]. Microarrays, combined with GenMAPP analysis of mouse lung tumor models, showed that multiple genes affected by herbal medicine ATB are members of different genetic pathways such as ubiquitin-proteasome, Notch, Ras-MAPK, and G13 pathways [89], which are important in mitogenesis, neoplastic transformation [93] and apoptosis [94]. This gene expression microarray study showed that ATB is a potential tumor suppressor capable of targeting cell proliferation, differentiation, and apoptosis [89].

The proteomic profile of MCF-7 cells treated with Zilongjin, an herbal antitumor medicine, showed the downregulation of HSP27, a blocker of apoptosis [95]. Zilongjin also suppressed the expression of eIF3I and eIF1AY proteins, which are important regulators of translation initiation [95]. Collectively, the proteomic approach can identify translational perturbations in cancer cells and protein-wide changes from these perturbations in response to stimuli. Microarray based gene expression analysis of four different lung cancer cell lines treated by Zilongjin showed that 170 genes were upregulated and 313 were downregulated by the drug. Of these 483 genes, eleven genes including *HELLS*, *JUN*, *XIAP*, *MCM6*, *CDKN2C*, *CCNE2*, *HN1L*, *TFDP2*, *CCNG2*, *GADD45A*, and *CDKN1A* were found to be involved in cancer-related pathways such as apoptosis, cell cycle, and MAPK cascade [96]. 

**Figure 1:**
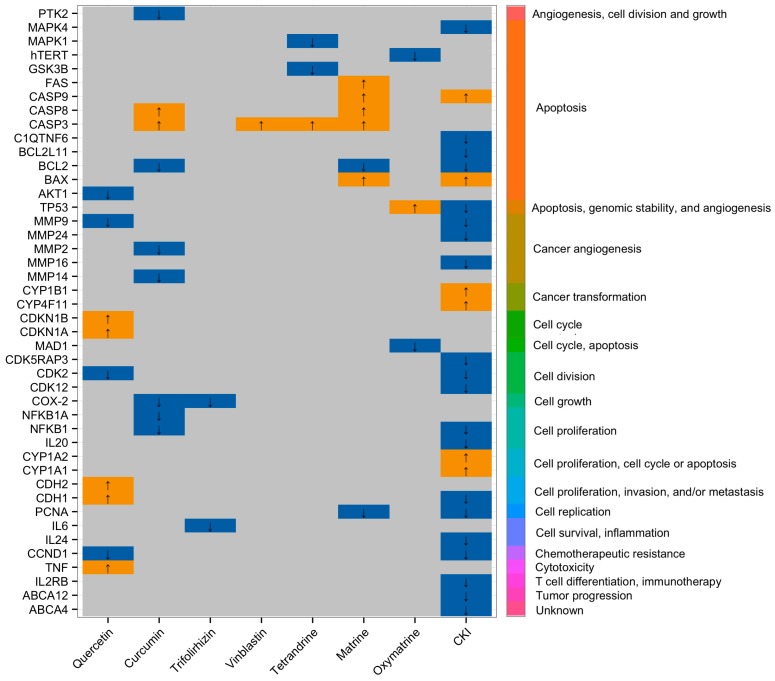
Differential gene expression in different cancer cell lines induced by flavonoids and alkaloids as well as Compound Kushen Injection (CKI). Left hand axis shows differentially expressed genes, bottom axis shows natural anti-cancer agent treatments (Quercetin, Curcumin, Trifolirhizin = flavonoids), (Vinblastine, Tetrandrine, Matrine, Oxymatrine = alkaloids), and (compound mixture = CKI) and the right hand axis shows Gene Ontology (GO) clustering and annotation of differentially expressed genes. Up and down-regulated genes (up = YELLOW and down = BLUE) known to be affected by natural compound anti-cancer agents were obtained from http://crdd.osdd.net/raghava/npact/browse.php and significantly differentially expressed genes from CKI treated MCF-7 cells were obtained from RNA-Seq experiments conducted by Qu et al. [87].

Taken together, the findings based on the molecular and ‘–omics approaches’ suggest that natural compound mixtures have multiple targets in cancer cells. Combined compounds from two or more sources are potential resources for the development of multi-targeted anti-cancer therapeutics and have a broader range of molecular targets in cancer cells. It is important to bear in mind that the clinical effectiveness of some of these combined drugs have only been reported in non-English language papers and these often lack compelling clinical data. Therefore, more work is needed to evaluate natural compound mixtures as cancer treatments. Figure 1 shows the molecular targets of two groups of single compounds, alkaloids and flavonoids, compared to a compound mixture, CKI, that contains both groups of natural compounds in an in vitro setting. Summaries of therapeutic effects from single or mixtures of natural compounds and their possible cellular mechanisms are shown in Table 1. 

## 5. Alternative Approach of Triggering Apoptosis Using Metal-Derivatized Natural Compounds

Structurally modified anti-cancer compounds can be highly effective in treating cancer due to their controlling chemo-, site- selectivity in cells. The heavy metal-based compound, cisplatin, has a broad spectrum of cytotoxicity that makes it among the most popular and effective chemotherapy drugs. Cisplatin and compounds of this type are used to treat approximately 50%–70% of all cancer patients [105]. Although metal-based anti-cancer drugs show significant effectiveness against cancer, they can also have severe adverse effects. Moreover, different mechanisms are used by cancer cells to resist cytotoxic drugs, complicating the development of novel potent anti-cancer drugs. Natural compounds have been shown to have low adverse reactions in normal cells of cancer patients, and natural compound-metal complexes have been confirmed as potential (pro) drugs [106]. The alkaloid liriodenine has anti-tumor activity and when combined with platinum (II) and ruthenium (II), the liriodenine-metal complexes covalently bind to DNA and enhance the cytotoxicity of liriodenine. When Gallium (III) and Tin (IV) are combined with matrine, these combinations further modulate cell cycle arrest at the G2/M phase, and Gold (III)-matrine complexes inhibit topoisomerase I [106], thereby causing DNA replication processes in cancer cells to malfunction. The actions of these different metal-based compounds vary depending on cell type. For instance, Gallium III-matrine and Gold III-matrine compounds have significant anti-proliferative activities in SW480 cells, HeLa cells, HepG2 cells, and MCF-7 cells, respectively [106]. It is worth noting that the efficacy of synthetic metal-natural compounds in vitro greatly exceeds those of cisplatin and matrine alone. These results indicate that metal-based cytotoxic natural compounds may hold promise as anti-cancer therapeutics, based on their multi-targeting effects on cancer cell regulatory networks. A summary of therapeutic effects by metal-derivatized natural compounds and their mechanisms of actions compared to their original forms and/or existing metal-based anticancer drugs are shown in Table 2. 

## 6. Chemoresistance in Cancer and Chemosensitization by Natural Compounds

The main reason for cancer treatment failure by chemotherapy is the emergence of drug-resistance during cancer progression. Collectively, MDR occurs when ABC transporters become overexpressed. Out of 48 human ABC transporters, the mechanisms of actions of P-gp, MDR1, ABCB1, MRP1/ABCC1, and ABCG2 have been widely reported clinically [113]. However, additional ABC transporters have been explored as targets for cancer therapeutics in drug discovery. For instance, four ABC transporter genes (*ABCA4*, *ABCC3*, *ABCC5*, and *ABCC8*) were upregulated in resistant MCF-7/AdVp3000 cells, whereas complete or partial downregulation of these genes was observed in the revertant MCF-7/AdVpRev cells [114]. Overexpression of drug resistance associated genes *ABCA4* [113,114] and *ABCA12* [115,116] were observed in human pancreatic cancers and MCF-7 breast cancer cell lines, respectively. Therefore, it might be productive to scrutinize the mechanisms of actions of less commonly studied ABC transporters from drug resistant cancer cell lines when treated with natural compounds. 

To overcome drug resistance, natural compounds such as sesquiterpenes, flavonoids, alkaloids, diterpenoids, saponins, and polyphenolic compounds [11,21] are substituted or applied in combination with existing drugs. These compounds are known to have anti-tumor activities and can not only kill cancer cells but also restore drug sensitivity. For example, tetrandrine (bioactive alkaloid) modulates P-gp-mediated drug efflux in vitro and has anti-neoplastic activity when given together with doxorubicin to mice bearing resistant MCF-1/DOX cells in vivo [13,14]. Another natural product is Quercetin (a flavonoid [11]), which blocks *MDR1* transcription, thereby suppressing P-gp expression [117] and restoring daunorubicin chemosensitivity in HL-60/DOX and K562/DOX cell lines [118,119]. 

A different flavonoid, curcumin, inhibits the main ABC transporters such as P-gp, MRP1, and ABCG2, and increases vincristine chemosensitivity in SGC7901/VCR cell lines [120,121]. 20(*S*)-Ginsenoside Rg3 (saponins) chemosensitizes vincristine, doxorubicin, etoposide, and colchicine resistant KBV20 cell lines in a time- and dose-dependent manner [11]. Oxymatrine chemosensitizes cisplatin resistant HeLa/DDP cells by suppressing inosine monophosphate dehydrogenase type II (IMPDH2) levels and inducing apoptosis through the mitochondrial pathway [54]. CKI may also act as a stimulator of drug resistance reversal and chemosensitization in cancer. Transcriptome analysis from Qu et al. revealed that two drug resistant ABC transporter genes, *ABCA12* and *ABCA4*, were significantly downregulated in CKI treated MCF-7 cells [87]. These results support the assertion that natural compounds can reverse or alter chemoresistance. 

## 7. Apoptosis in MDR Cells through Modulation of MicroRNA (miRNA) Networks by Natural Compounds

The compelling link between possible successful cancer chemotherapy and triggering apoptosis by chemosensitization is connected in this review by the involvement of microRNAs. miRNAs are highly conserved small non-coding RNA molecules that directly interact with their target mRNA, causing either mRNA transcriptional degradation or translational repression that results in the reduction of gene expression [122]. miRNAs have emerged as both potential therapeutic agents and biomarkers [123]. Some have oncogenic properties while others act as tumor suppressors. Miller et al. reported that eight upregulated miRNAs and seven downregulated miRNAs were found in tamoxifen resistant human breast cancer MCF-7 cells [124]. Moreover, increased expression of miR-415 is correlated with the decreased expression of *MDR1* genes and seems to elevate sensitivity to doxorubicin in DOX-resistant breast cancer cells [125]. In tumors, epithelial-to-mesenchymal transition (EMT) plays an important role of tumor invasion/migration and metastasis [126]. ZEB1 and ZEB2 repress E-cadherin expression [127,128] and downregulation of E-cadherin allows epithelial cells to undergo EMT, which contributes resistance to EGFR-directed therapy in cancer. Members of the miR-200 family, particularly miR-200c and miR-200b, target ZEB1 and ZEB2 and control EMT to sensitize EGFR therapy [129].

**Figure 2:**
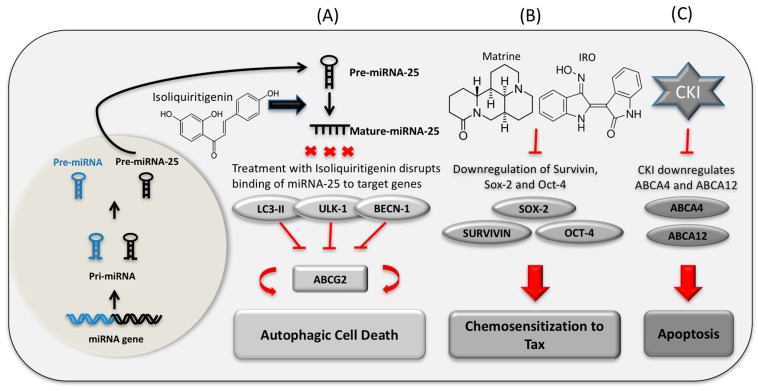
Three panels (**A**–**C**) show the influence of natural compounds on gene expression in terms of MDR regulation. (**A**) Treatment of Isoliquiritigenin blocks binding of autophagy-related miR-25 to the 3′ UTR region of *ULK-1*, *LC3-II*, and *BECN-1* in killing drug-resistant breast cancer cells [133]; (**B**) Matrine and indirubin-3′-monoxime (IRO) reverse chemoresistance of paclitaxel (TAX) by downregulating the expression of Sox-2, Survivin, and Oct-4 proteins in human squamous cell carcinoma [134]; (**C**) CKI suppresses the expression of the drug resistant related genes *ABCA12* and *ABCA4* in cancer cell apoptosis. The involvement of miRNAs in the regulation of these genes with respect to drug resistance has not yet been confirmed [87].

Growing evidence indicates that natural compounds are important regulators of miRNA mediated genetic pathways in cancer. For example, matrine significantly reduces the overexpression of miRNA-21 and stimulates apoptosis in HepG2 and Hep3B cells [130]. Matrine also downregulated 14 miRNAs and upregulated their target genes in the MAPK signaling pathway in SGC7901 human gastric cancer cells [131]. Another natural compound, oxymatrine, acts on human ovarian cancer OVCAR3 cells by upregulating miR‑29b, which downregulates the expression level of matrix metalloproteinase‑2 and induces apoptosis [132]. In the microarray analysis of MCF-7 cells, upregulation of 45 miRNAs was shown after treatment with TCM Aidi injection. Of these, miRNA-126 was found to be a suppressor of proliferation of MCF-7 cells [104]. Research showed that inhibition of miRNA-25 by a natural phenol, isoliquiritigenin, leads to autophagy in MCF-7/ADR cells via increased expression of autophagy regulator ULK1 [133]. In the transcriptome of CKI treated MCF-7 cells, the upregulation of hsa-miR-6879 and downregulation of its target drug resistant gene *ABCA12* were observed [87]. However, the prediction of the role of pre-miR-6879 in terms of the regulation of ABC transporters still needs to be experimentally confirmed. The link between natural compounds inducing miRNA mediated drug resistance reversal and chemosensitization, leading to cancer cells autophagy and apoptosis, supports the investigation of natural compounds in drug resistant cancer chemosensitization therapy. Whilst it is known that miRNAs are important in phytochemically mediated cancer cell death, little is known about full or partial reversal of MDR via the regulation of miRNAs by natural compounds. Hence, there is a gap between the use of natural compounds that regulate miRNA and MDR that is not well understood. A model of the regulatory interactions between drugs, miRNA, and target genes in the context of autophagy, drug resistance reversal, and apoptosis is shown in Figure 2. 

## 8. Conclusions

Despite significant progress in the fields of cancer diagnosis and chemotherapy, cancer remains one of the greatest causes of death worldwide. Novel approaches to cancer management often fail due to frequent genetic alterations and mutations in cancer genomes. Because of the high frequency of side effects caused by chemotherapy, metastatic cancers still need new, more effective chemotherapeutics. There is emerging interest in developing drugs to tackle these problems by using natural compounds, which may affect multiple targets with lower side effects and be effective against several cancer types. Despite attempts in chemotherapy using natural compounds, many questions remain regarding their efficacy and potential modes of action. From the medicinal chemistry point of view, an important fact when applying the natural compounds to consider is the impurities of the extracted herbal medicine that have their own biological activity which may provide false positive signal of the molecules during drug screening. In addition, flawed scientific evidence from natural product mixtures can suggest false positive therapeutic benefits. Therefore, careful examination to approve the molecular target engagement while researching drug discovery is necessary. The best possible drug combinations are based on the understanding of the cancer-specific context of mutated oncogenes, tumor suppressor genes, and their regulatory pathways. To evaluate novel treatment approaches involving the use of mixtures of natural compounds, we must understand how coding and non-coding RNAs, oncogenes, downregulated tumor suppressor genes, and mutated genes, as well as their signal transduction pathways, respond to these drugs. Systems biology can help characterize the roles of functionally cryptic elements such as long and short ncRNAs in cancer. We have reviewed the effectiveness of natural compounds in cancer cells in terms of triggering apoptosis and chemosensitization through the application of molecular genetic and “-omics” approaches. There is evidence to suggest that natural compounds might be effective and less toxic in some circumstances. Furthermore, metal-derivatized natural compounds can also trigger apoptosis and natural compound-metal complexes have been confirmed as potential (pro) drugs [106]. This suggests that natural compounds and their derivatives merit further investigation as anti-cancer therapeutics. 

The effect of multi-drug resistance during cancer progression is another important hurdle for chemotherapy. In resistance to chemotherapy-induced cancer cell death, genetic alterations in the drug-induced apoptotic program also cause MDR to biochemically unrelated drugs [135]. This is illustrated by the relationship between chemoresistance and targeted upregulation of apoptosis regulator *bcl-2* by multiple DNA damaging stimuli [135]. Therefore, it is important to study the role of natural compounds in chemosensitization, since this may create new avenues for novel drug development in cancer treatment. The investigation of natural compound mixtures as apoptosis-inducing and chemosensitizing cancer therapeutics will improve our understanding of the molecular changes in cancer cells and should provide clues about how the disease can be controlled. Our current knowledge of natural compounds’ effects on cancer is mainly from cell-based experiments and partly from in vivo experiments. To date, the clinical effectiveness of some of these combined drugs has not been robustly demonstrated. As a result, additional research using in vivo systems and better clinical trials is needed to determine the safety and clinical usefulness of these drugs. 

## Figures and Tables

**Table 1:** Reported therapeutic effects by single or mixtures of natural compounds in different stages of cellular mechanisms.

Ref.	Herbal Medicines	Types of Cancer	Cell Lines/Model	Mechanisms of Actions
[97]	Curcumin	Colorectal	Colorectal cancer stem cells (CCSCs)	Apoptosis
[98]	Ginsenoside Rg3	Liver	Tumor bearing rats	Apoptosis, Immune responses
[99]	Curcumin	Breast	MCF-7	Anti-inflammation
[100]	Matrine	Lung	HepG2	Proliferation and metastasis chemosensitization
[72]	Quercetin	Lung	HepG2	Apoptosis
[95]	Zilongjin	Breast	MCF-7	Inhibits malignant proliferation, apoptosis
[101]	Triterpenes from *Ganoderma lucidum*	Cervical	HeLa	Cell death, oxidative stress, calcium signaling, and ER stress
[64]	Curcumin	Breast	MCF-7	Apoptosis
[102]	Triterpenes from *Patrinia heterophylla*	Leukemia	K562	Energy metabolism, oxidative stress, signal transduction, differential induction, protein biosynthesis, and apoptosis
[54]	Oxymatrine	Cervical	HeLa	Inhibits proliferation, apoptosis
[103]	Sanguinarine from Papaveraceae family	Pancreatic	BxPC-3, MIA PaCa-2	Decreases cellular hypoxia and cell proliferation, induces apoptosis leading to cancer cells inhibition
[89]	Zeng Sheng Ping (Antitumor B)	Lung	Mouse lung	Ubiquitin-proteasome, Notch, Ras-MAPK, G13 pathway, cell proliferation, differentiation, and apoptosis
[104]	Aidi injection	Breast	MCF-7	Inhibits proliferation, apoptosis
[96]	Zilongjin	Lung	A549, H446, H460, and H520	Cell cycle regulation, MAPK cascade, and apoptosis
[87]	Compound Kushen Injection	Breast	MCF-7	Cell cycle regulation, cell growth related pathway

**Table 2:** Mechanisms of action of metal-derivatized natural compounds compared to their original forms and/or existing metal-based anticancer drugs.

Groups of Natural Compounds	Metal Derivatized Natural Compounds	Source	Cell Lines/Model	Mechanisms of Actions	Remarks	Ref.
**Alkaloids**	GL331	**Compound**: Podophyllotoxin **Plant**: *Podophyllum* species	KB/VCR, MCF-7/ADR, and HL60/VCR	TOPO II inhibitor, cell cycle arrest at G2, cause DNA breakage and apoptosis via inhibiting protein tyrosine kinase	GL331 shows greater cytotoxicity in vitro and in vivo, and overcomes multi-drug resistance (MDR) compared to etoposide. GL331 is now in phase II clinical trial	[107]
	[H-MT][GaCl_4_] [H-MT][AuCl_4_] [Sn(H-MT)Cl_5_]	**Compound**: *MT* (*Matrine*) **Plant**: *Sophora flavescens*	SW480, HeLa, HepG2, and MCF-7	Cell cycle arrest at the G_2_/M phase	MT + Gallium (GaCl_4_) and MT + Gold (AuCl_4_) enhanced the cytotoxicity better than MT alone and cisplatin	[108]
	[Ru(N–N)_2_ (Norharman)_2_] (SO_3_CF_3_)_2_	**Compound**: Norharman **Plant**: *Peganum harmala* L.	HepG2, HeLa, MCF-7, and MCF-10A	Cell cycle arrest at G0/G1, apoptosis via mitochondrial dysfunction and ROS accumulation	IC_50_ value of the complex is much lower and the anti-proliferative activity is much higher than those of Norharman and cisplatin	[109]
	[L+H][AuCl_4_] [AuCl_3_ L]	**Compound**: Liriodenine (L) **Plant**: *Zanthoxylum nitidum*	MCF-7	TOPO I inhibitor, cell cycle arrest at S phase	Higher anti-proliferative activity than cisplatin, Adriamycin, liriodenine alone, and NaAuCl4	[110]
**Flavonoids**	hesperetin [CuL_2_(H_2_O)_2_]nH_2_O	**Compound**: hesperetin, **Plant**: *Stilbella fimetaria*	HepG2, and SGC-7901	Growth inhibition	DNA binding affinity of hesperetin-Cu(II) complex is stronger than that of free hesperetin	[111]
	Zn(morin)_2_.3H_2_O Cu(morin)_2_.2H_2_O	**Compound**: Morin **Plant**: *Maclura pomifera*	Hep-2, BBHK-2, BHK21, and HL-60	In vitro antitumor activity	Higher anti-proliferative activity than morin alone	[106]
	Cu(Que)_2_(H_2_O)_2_	**Compound**: quercetin, **Plant**: various fruits and vegetables	A549	DNA breakage, apoptosis via generation of ROS and intercalation into DNA	Higher cytotoxic activity than that of quercetin alone	[112]

## Abbreviations

CKI	Compound Kushen Injection
MDR	Multi-drug Resistance
ATB	Antitumor B
NPACT	Naturally Occurring Plant-based Anti-cancer Compound-Activity-Target Database
TCM	Traditional Chinese Medicine
QoL	Quality of Life
MT	Matrine
GO	Gene Ontology
IRO	Indirubin-3′-monoxime
CS-IVa-Be	Chikusetsusaponin IV a Butyl Ester
VEGF	Vascular Endothelial Growth Factor
Akt	Protein Kinase B
NF-κB	Nuclear Factor κ-light-chain-enhancer of activated B cells
ER	Endoplasmic Reticulum
MAPK	Mitogen Activated Protein Kinase
